# ADP-ribosylation Factor-related Protein 1 Interacts with NS5A and Regulates Hepatitis C Virus Propagation

**DOI:** 10.1038/srep31211

**Published:** 2016-08-23

**Authors:** Yun-Sook Lim, Huong T. T. Ngo, Jihye Lee, Kidong Son, Eun-Mee Park, Soon B. Hwang

**Affiliations:** 1 National Research Laboratory of Hepatitis C Virus and Ilsong Institute of Life Science, Hallym University, Anyang, South Korea; 2 Korea National Institute of Health, Cheongwon-gun, South Korea

## Abstract

The life cycle of hepatitis C virus (HCV) is tightly coupled to the lipid metabolism of host cells. In order to identify host factors involved in HCV propagation, we have previously screened a small interfering RNA (siRNA) library targeting host genes that control lipid metabolism and lipid droplet (LD) formation using cell culture-grown HCV (HCVcc)-infected cells. In this study, we selected and characterized the gene encoding ADP-ribosylation factor-related protein 1 (ARFRP1). ARFRP1 is essential for LD growth and is involved in the regulation of lipolysis. siRNA-mediated knockdown of ARFRP1 significantly inhibited HCV replication in both subgenomic replicon cells and HCVcc-infected cells. ARFRP1 interacted with NS5A and NS5A partially colocalized with LD. Silencing of ARFRP1 abrogated HCV-induced LD growth and viral protein expressions. Moreover, ARFRP1 recruited synaptosomal-associated protein 23 (SNAP23) to sites in close proximity to LDs in HCV-infected cells. Silencing of ARFRP1 ablated relocalization of SNAP23 to LD. These data indicate that HCV regulates ARFRP1 for LD growth to facilitate viral propagation and thus ARFRP1 may be a potential target for antiviral therapy.

Hepatitis C virus (HCV) is an enveloped virus with a single-stranded, positive-sense RNA virus that belongs to the *Hepacivirus* genus in the *Flaviviridae* family^1^. Currently, approximately 170 million people are chronically infected with HCV worldwide^2^. HCV is the leading cause of liver fibrosis, liver cirrhosis, and hepatocellular carcinoma. HCV RNA encodes a single polyprotein that is cleaved by both cellular and viral proteases into 10 mature viral proteins, including structural (core, E1, E2) and nonstructural (p7 and NS2 to NS5B) proteins^3^. There is no prophylactic vaccine for HCV. Currently, various direct-acting antivirals (DAAs) in combination with pegylated interferon and ribavirin are available to treat HCV patients. However, these DAAs still show genotypic differences in cure rate and occasional occurrence of resistance-associated variants. Furthermore, these drugs are too burdensome and hence unaffordable for most HCV patients worldwide. Therefore, development of novel class of host-targeted antivirals may be an alternative strategy to develop broadly active and reasonable antivirals in the future.

HCV appropriates host cell lipid droplet (LD) for production of infectious virus particles^4^. Therefore, the life cycle of HCV is tightly linked to lipid metabolism and LDs of host cells. LD is an organelle that contains a core of neutral lipids surrounded by a monolayer of amphipathic lipids and perilipin, adipocyte-differentiation-related protein (ADRP), and tail-interacting protein 47 (TIP47) proteins^5^,^6^. Many cellular proteins participate in the turnover, formation, fusion, and trafficking of LDs^5^,^6^,^7^. LDs are dynamic organelles that not only involved in cellular processes^5^ but also required for the propagation of Flavivirus^8^,^9^,^10^. Chronic HCV infection often causes steatosis and abnormal lipid metabolism that may be linked to enhanced LD formation^11^. HCV-induced steatosis is associated with changes in cellular cholesterol and lipid metabolism^12^,^13^,^14^,^15^. Therefore, understanding the molecular mechanisms underlying biogenesis, growth, maintenance, and degradation of LD will provide clues for treatment of metabolic diseases and virus-mediated pathogenesis^16^.

ADP-ribosylation factor (ARF)-related protein 1 (ARFRP1), also known as ARP^17^, is a membrane-associated 25-kDa GTPase. Knockout of ARFRP1 gene in mice resulted in embryonic lethality and apoptosis in ectodermal cells^18^. ARFRP1 is implicated in the membrane trafficking between the trans-Golgi network and other membrane organelles^19^,^20^,^21^. Moreover, ARFRP1 is essential for cell survival^18^ and also regulates the growth of LDs^7^,^22^. In the present study, we demonstrated that silencing of ARFRP1 impaired HCV RNA and protein expressions, and subsequent HCV infectivity. Moreover, knockdown of ARFRP1 significantly reduced HCV-mediated LD growth. We further showed that SNAP23 protein, a downstream effector of ARFRP1 which has been known to be required for LD assembly, was also required for HCV production. Overall, our study provides the first evidence that HCV regulates ARFRP1 together with SNAP23 for LD growth to facilitate viral propagation.

## Results

### ARFRP1 is required for HCV propagation

**Figure 1:**
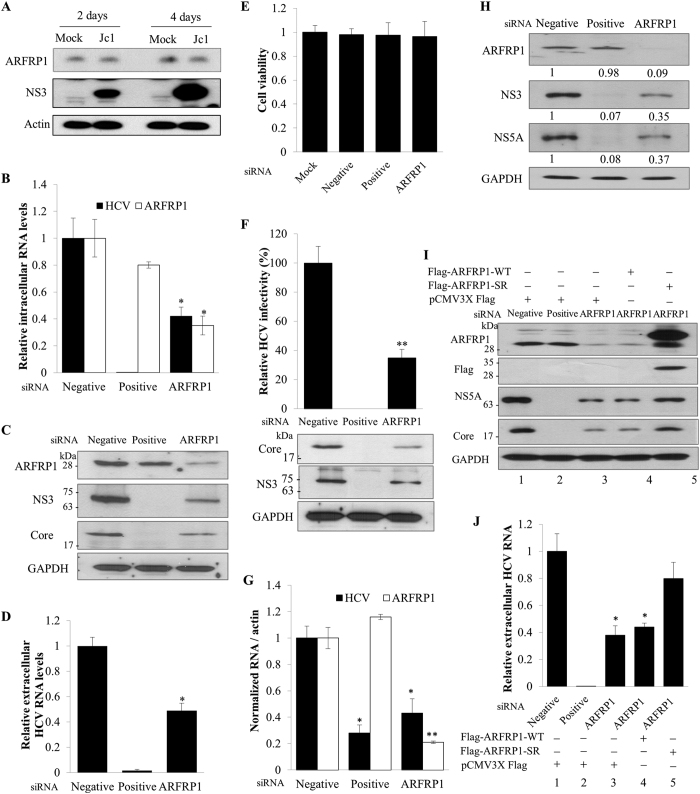
ARFRP1 is required for HCV propagation. (**A**) Huh7.5 cells were either mock-infected or infected with HCV Jc1 for 4 h. Total cellular lysates harvested at the indicated time intervals were immunoblotted with the indicated antibodies. (**B**) Huh7.5 cells were transfected with 20 nM of the indicated siRNAs. At 2 days after siRNA transfection, cells were infected with Jc1 for 4 h. At 48 h postinfection, intracellular RNA levels of both HCV and ARFRP1 were quantified by qRT-PCR. Negative, scrambled siRNA; positive, HCV-specific siRNA. The asterisk indicates significant difference (*p < 0.05) from the value for the negative control. (**C**) Huh7.5 cells transfected with the indicated siRNAs were infected with Jc1. Total cell lysates harvested at 48 h postinfection were immunoblotted with the indicated antibodies. (**D**) Extracellular RNAs isolated from the culture supernatant were quantified by qRT-PCR. (**E**) Huh7.5 cells were transfected with 50 nM of the indicated siRNAs for 96 h and then cell viability was assessed by MTT assay. (**F**) Naïve Huh7.5 cells were infected with virus-containing culture supernatants harvested from (**D**). HCV infectivity was determined by a focus-forming assay (upper panel) and total cell lysates were immunoblotted with the indicated antibodies (lower panel). (**G**) HCV subgenomic replicon cells were transfected with the indicated siRNAs. At 72 h after siRNA transfection, intracellular RNA levels of both HCV and ARFRP1 were quantified by qRT-PCR. The asterisks indicate significant differences (*p < 0.05; **p < 0.01) from the value for the negative control. (**H**) HCV subgenomic replicon cells were transfected with the indicated siRNA constructs. Total cell lysates harvested at 72 h after transfection were immunoblotted with the indicated antibodies. The band intensity was quantified using ImageJ software. (**I**) Huh7.5 cells transfected with the indicated siRNAs were infected with Jc1 for 4 h and then further transfected with the indicated combinations of plasmids. At 48 h after transfection, cell lysates were immunoblotted with the indicated antibodies. (**J**) Huh7.5 cells were treated as described in legend to Fig. 1H and then extracellular RNAs were quantified by qRT-PCR. Flag-ARFRP1-WT, Flag-tagged wild-type ARFRP1; Flag-ARFRP1-SR, Flag-tagged siRNA-resistant mutant ARFRP1. All experiments were performed in duplicate.

To identify host factors involved in HCV propagation, we have previously screened a siRNA library targeting 114 host genes that might control lipid metabolism and LD formation using HCVcc-infected cells. From these siRNA pools, 10 host genes were identified as candidate hits^23^. Of these, we selected and characterized the gene encoding ARFRP1 since this gene has been implicated in cell survival and regulation of LD growth^6^,^22^. We first determined whether protein expression level of ARFRP1 was changed over time after HCV infection. As shown in Fig. 1A, viral protein expression level was increased gradually during HCV infection. However, protein expression level of ARFRP1 was not affected by HCV infection. To investigate the functional involvement of ARFRP1 in HCV propagation, Huh7.5 cells were transfected with the indicated siRNAs and then infected with Jc1. Silencing of ARFRP1 expression led to significant reduction in intracellular HCV RNA (Fig. 1B) and protein (Fig. 1C) levels. Consistently, extracellular HCV RNA level was also significantly decreased in ARFRP1 knockdown cells (Fig. 1D) with no effect on cell viability (Fig. 1E). To confirm this result, culture supernatant harvested from cells treated as described in Fig. 1D were used to infect naïve Huh7.5 cells and HCV infectivity was determined. As shown in Fig. 1F, HCV infectivity was significantly decreased in ARFRP1 knockdown cells as compared to control siRNA-treated cells (upper panel). Similarly, HCV protein levels were also markedly reduced in ARFRP1 knockdown cells (Fig. 1F, lower panel). To further explore the possible role of ARFRP1 in HCV RNA replication, Huh7 cells harboring HCV subgenomic replicon were transfected with the indicated siRNAs. As expected, silencing of ARFRP1 expression led to significant decrease in RNA (Fig. 1G) and protein (Fig. 1H) levels of HCV in replicon cells. We also verified that cell viability was unaffected in ARFRP1-knockdown HCV replicon cells (data not shown). To exclude the off-target effect of ARFRP1 siRNA, we generated a siRNA-resistant mutant ARFRP1. As shown in Fig. 1I, exogenous expression of the siRNA-resistant mutant ARFRP1, but not of wild-type ARFRP1, rescued HCV protein level (lane 4 versus lane 5) in ARFRP1-enervated cells. Consistently, exogenous expression of the siRNA-resistant mutant ARFRP1 recovered extracellular HCV RNA level (Fig. 1J, lane 4 versus lane 5) in ARFRP1-knockdown cells. All these data suggest that ARFRP1 is required for HCV propagation.

### Overexpression of ARFRP1 increases extracellular HCV RNA but not HCV protein level

**Figure 2:**
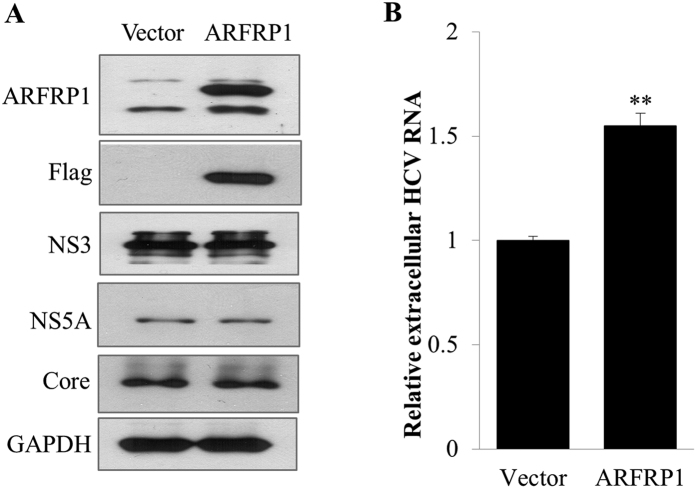
Overexpression of ARFRP1 increases extracellular HCV RNA level. (**A**) Huh7.5 cells were infected with Jc1 for 48 h and then transiently transfected with either empty vector or Flag-tagged ARFRP1 expression plasmid. Total cell lysates harvested at 48 h after transfection were immunoblotted with the indicated antibodies. (**B**) Huh7.5 cells were treated as described in (**A**) and extracellular HCV RNAs were quantified by qRT-PCR. Experiments were performed in duplicate. Error bars indicate the standard deviations of the means. The asterisks indicate a significant difference (**p < 0.01).

Since knockdown of ARFRP1 impaired HCV propagation, we explored the overexpression effect of ARFRP1 on HCV propagation. For this purpose, Huh7.5 cells infected with Jc1 were transiently transfected with either empty vector or Flag-tagged ARFRP1 plasmid. At two days after transfection, both protein expression and extracellular RNA levels of HCV were determined. As shown in Fig. 2A, overexpression of ARFRP1 displayed no effect on HCV protein levels. However, extracellular HCV RNA level was significantly increased in cells overexpressing the ARFRP1 protein (Fig. 2B). These data suggest that ARFRP1 may also be involved in assembly or release step of HCV production.

### ARFRP1 interacts with NS5A protein

**Figure 3:**
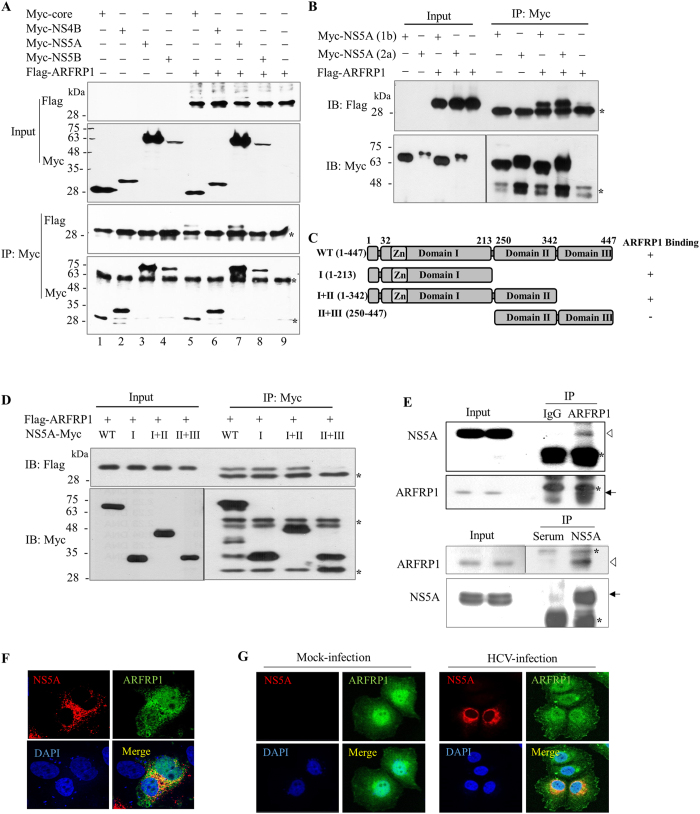
ARFRP1 specifically interacts with NS5A. (**A**) HEK293T cells were transfected with the indicated combinations of plasmids. At 48 h after transfection, cell lysates were immunoprecipitated with an anti-Myc monoclonal antibody, and then bound proteins were immunoblotted by using an anti-Flag antibody (lower, top panel). Immunoprecipitation efficiency of each viral protein was verified by immunoblotting with an anti-Myc antibody (lower, bottom panel). IP, immunoprecipitation; IB, immunoblot. Input proteins were verified using indicated antibodies (upper panels). Asterisks indicate the heavy and light chain IgG. All experiments were carried out in duplicate. (**B**) HEK293T cells were cotransfected with Flag-tagged AFRRP1 and Myc-tagged NS5A (genotype 1b or 2a). At 48 h after transfection, cell lysates were immunoprecipitated with an anti-Myc antibody, and then bound proteins were detected by immunoblot analysis using an anti-Flag antibody (upper panel). Protein expressions of Myc-tagged NS5A (lower, left panel) and immunoprecipitation efficiency (lower, right panel) were verified using an anti-Myc antibody. (**C**) Schematic illustration of both wild-type and mutant constructs of NS5A plasmid. (**D**) HEK293T cells were cotransfected with the indicated combinations of expression plasmids. At 48 h after transfection, cell lysates were immunoprecipitated with an anti-Myc antibody, and bound proteins were immunoblotted with an anti-Flag antibody (upper, right). Both protein expressions and immunoprecipitation efficiency were verified by immunoblotting with the indicated antibodies (lower panels). (**E**) (Upper, top panel) Huh7.5 cells infected with Jc1 were immunoprecipitated with either IgG or an anti-ARFRP1 antibody, and bound protein was immunoblotted with rabbit anti-NS5A antibody. Arrowhead indicates NS5A and arrow denotes ARFRP1. (Lower, top panel) The same cell lysates were immunoprecipitated with either control rabbit serum or rabbit anti-NS5A antibody, and bound protein was immunoblotted with an anti-ARFRP1 antibody. Arrowhead indicates ARFRP1 and arrow denotes NS5A protein. (**F**) Huh7.5 cells were transfected with Flag-tagged ARFRP1 plasmid and then infected with Jc1. Immunofluorescence staining was performed by using rabbit anti-NS5A antibody and TRITC-conjugated goat anti-rabbit IgG to detect NS5A (red), and an anti-Flag antibody and FITC-conjugated goat anti-mouse IgG to detect ectopic expression ARFRP1 (green). Cells were counterstained with DAPI to label nuclei (blue). (**G**) Huh7.5 cells were either mock-infected or infected with Jc1. Immunofluorescence staining was performed by using rabbit anti-NS5A antibody and TRITC-conjugated goat anti-rabbit IgG (red), and an monoclonal anti-ARFRP1 antibody and FITC-conjugated goat anti-mouse IgG to detect endogenous ARFRP1 (green).

Next, we asked whether functional involvement of ARFRP1 in HCV propagation was mediated through virus-host protein interaction. For this purpose, HEK293T cells were cotransfected with Flag-tagged ARFRP1 and each of Myc-tagged HCV protein expression plasmid as indicated. At 48 h after transfection, total cellular proteins were immunoprecipitated with an anti-Myc antibody and then bound proteins were immunoblotted with an anti-Flag antibody. As shown in Fig. 3A, ARFRP1 interacted with both core and NS5A protein. It was noteworthy that ARFRP1 strongly interacted with NS5A (Fig. 3A, lane 7), whereas its binding with core protein was very weak (Fig. 3A, lane 5). We further confirmed that ARFRP1 binds to NS5A stronger than HCV core protein (Fig. S1). Therefore, we selected NS5A and further characterized protein interplay between ARFRP1 and NS5A. For this purpose, HEK293T cells were cotransfected with Flag-tagged ARFRP1 and Myc-tagged NS5A derived from either genotype 1b or genotype 2a. Total cell lysates harvested at 48 h after transfection were immunoprecipitated with an anti-Myc antibody and bound proteins were immunoblotted with an anti-Flag antibody. As demonstrated in Fig. 3B, ARFRP1 specifically interacted with NS5A derived from both genotype 1b and 2a. To determine the region in NS5A responsible for ARFRP1 binding, HEK293T cells were cotransfected with Flag-tagged ARFRP1 and various deletion mutants of NS5A (Fig. 3C), and protein interaction was determined by a transfection-based coimmunoprecipitation assay. As shown in Fig. 3D, ARFRP1 interacted with the mutant encompassing domain I (I) and the mutant harboring both domains I and II of NS5A (I+II). However, ARFRP1 was unable to bind the mutant lacking domain I of NS5A (II+III). This result indicated that domain I of NS5A was required for binding with ARFRP1. Next, we investigated whether endogenous ARFRP1 interacted with NS5A. Cell lysates harvested at 4 days postinfection were immunoprecipitated with either control IgG or an anti-ARFRP1 antibody, and bound protein was analyzed by immunoblotting with rabbit anti-NS5A antibody. As shown in Fig. 3E, endogenous ARFRP1 interacted with NS5A (upper panel). By reciprocal immunoprecipitation assay, we further confirmed that endogenous ARFRP1 interacted with NS5A in HCV-infected cells (Fig. 3E, lower panel). These data suggest that ARFRP1 may colocalize with NS5A protein. To investigate this possibility, Huh7.5 cells were transfected with Flag-tagged ARFRP1 and then infected with Jc1. At 36 h postinfection, subcellular localizations of ectopically expressed ARFRP1 and NS5A were analyzed by confocal microscopy. Figure 3F shows that ARFRP1 was widely expressed in both the nucleus and the cytoplasm in hepatoma cells and the cellular localization of ARFRP1 was unaffected by NS5A protein. Dual staining showed colocalization of ARFRP1 with NS5A in the cytoplasm as yellow fluorescence. We further verified that the cellular distribution of endogenous ARFRP1 was not altered by HCV infection, and both ARFRP1 and NS5A were colocalized in the cytoplasm in HCV-infected cells (Fig. 3G). Collectively, these data suggest that ARFRP1 specifically interacts with NS5A both *in vitro* and *in vivo*.

### ARFRP1 is involved in the regulation of LD growth in HCV-infected cells

**Figure 4:**
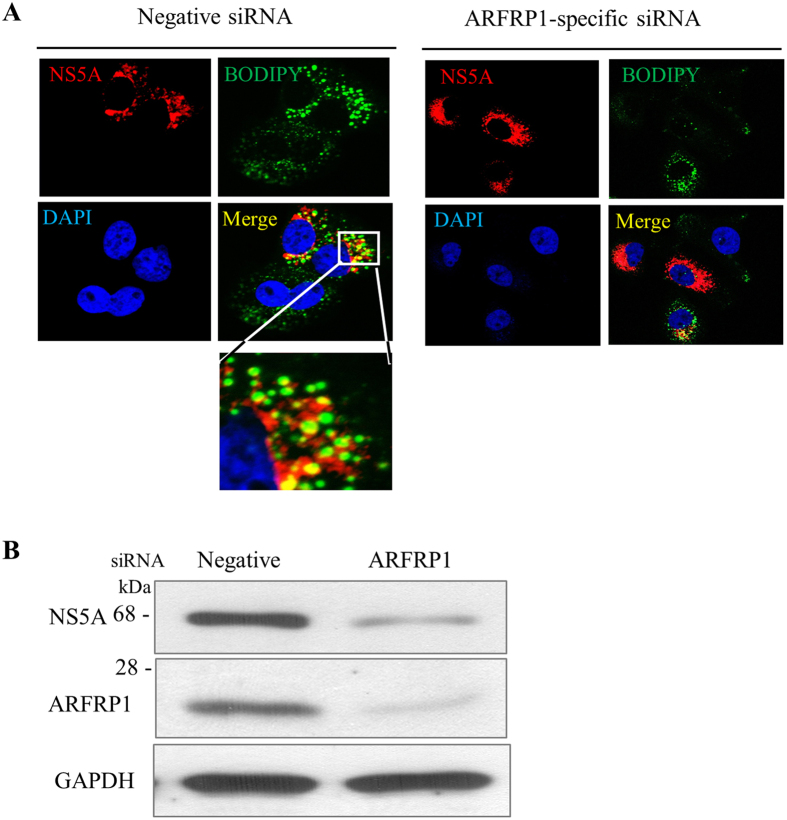
ARFRP1 is involved in the regulation of LD growth in HCV infected cells. (**A**) Huh7.5 cells were seeded on cover slides, transfected with either negative siRNA or ARFRP1-specific siRNA for 48 h, and then infected with Jc1. At 48 h postinfection, cells were fixed in 4% paraformaldehyde, and immunofluorescence staining was performed by using a rabbit anti-NS5A antibody and TRITC-conjugated donkey anti-rabbit IgG to detect NS5A (red). Cells were further incubated with 1 μM of BODIPY (439/503) (Invitrogen) to detect LDs (green). Dual staining showed colocalization of NS5A and LDs as yellow fluorescence in the merged image. The boxed area in the merged image is enlarged at the bottom. Cells were counterstained with DAPI to label nuclei (blue). (**B**) Huh7.5 cells were treated as described in (**A**) and total cell lysates were immunoblotted with the indicated antibodies.

Since ARFRP1 is implicated in the regulation of LD growth^6^,^22^, we investigated the role of ARFRP1 on LD growth in HCV-infected cells. Huh7.5 cells transfected with either negative siRNA or siRNA targeting ARFRP1 were infected with Jc1 and immunofluorescence staining was performed with the indicated antibodies. Consistent with previous reports^24^,^25^, LDs were accumulated in both HCV replicon and HCV infected cells (data not shown). We showed that NS5A partially colocalized with LDs in negative siRNA-transfected cells (Fig. 4A, left panel). It was noteworthy that silencing of ARFRP1 abrogated HCV-induced LD growth (Fig. 4A, right panel) and impaired HCV protein expression (Fig. 4B). Collectively, these data demonstrate that HCV regulates ARFRP1 for LD growth.

### ARFRP1 recruits SNAP23 to sites in close proximity to LDs in HCV-infected cells

**Figure 5:**
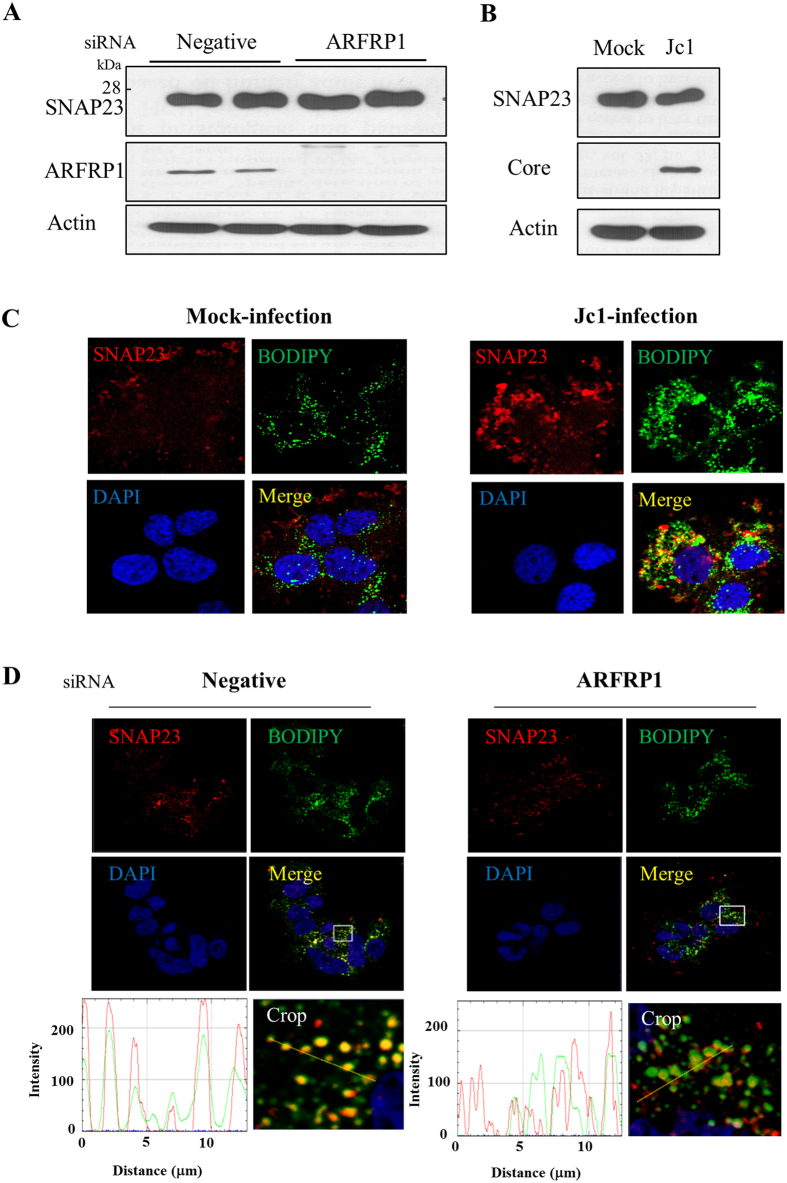
ARFRP1 recruits SNAP23 to sites in close proximity to LDs in HCV infected cells. (**A**) Huh7.5 cells were transfected with either negative siRNA or ARFRP1-specific siRNA constructs. At 48 h after transfection, cell lysates were immunoblotted with the indicated antibodies. Experiments were performed in duplicate. (**B**) Huh7.5 cells were either mock infected or infected with Jc1 for 4 h. At 2 days postinfection, total cell lysates were immunoblotted with the indicated antibodies. (**C**) Huh7.5 cells were either mock-infected or infected with Jc1 for 4 h. At 48 h postinfection, cells were fixed in 4% paraformaldehyde and immunofluorescence staining was performed by using rabbit anti-SNAP23 monoclonal antibody and TRITC-conjugated donkey anti-rabbit IgG to detect SNAP23 (red). Cells were further incubated with 1 μM of BODIPY (439/503) to detect LDs (green). Dual staining showed that a fraction of SNAP23 colocalized with LDs as yellow fluorescence in the merged image. Cells were counterstained with DAPI to label nuclei (blue). (**D**) Huh7.5 cells were infected with Jc1. After 24 h postinfection, cells were transfected with 20 nM of negative siRNA or ARFRP1-specific siRNA. At 3 days after transfection, cells were fixed and immunofluorescence staining was performed as described in (**C**). Immunofluorescence images were processed using the Zeiss LSM 700 laser confocal microscopy system. Colocalization of SNAP23 with LDs was analyzed by determining the distance and intensity of SNAP23 and BODIPY at the indicated yellow line in each crop by using Image.

It has been reported previously that SNAP23 is involved in LD fusion and functions as a downstream effector of ARFRP1^22^,^26^. SNAP23 protein is an essential component of the membrane fusion machinery and an important regulator of vesicle transport^26^,^27^. Since ARFRP1 was involved in the accumulation of LDs in HCV-infected cells, we speculated that ARFRP1 might modulate SNAP23 for the LD growth. We first investigated whether SNAP23 protein level was altered in either ARFRP1-knockdown cells or HCV-infected cells. As shown in Fig. 5A, silencing of ARFRP1 displayed no effect on SNAP23 protein expression. We also showed that SNAP23 protein expression level was not altered in Jc1-infected cells as compared with mock-infected cells (Fig. 5B). Interestingly, SNAP23 relocalized to sites in close proximity to LDs and partially colocalized with LDs in Jc1-infected cells but not in mock-infected cells (Fig. 5C). We further showed that SNAP23 was relocalized to sites in close proximity to LDs in Huh7 cells harboring HCV subgenomic replicon (Fig. S2). To further investigate the effect of ARFRP1 on SNAP23 localization, Huh7.5 cells infected with Jc1 were transfected with either negative siRNA or ARFRP1-specific siRNA and then stained with an anti-SNAP23 antibody and BODIPY. As shown in Fig. 5D, colocalization of SNAP23 with LDs was markedly reduced in ARFRP1-knockdown cells as compared with negative siRNA-treated cells, indicating that ARFRP1 was involved in protein relocalization of SNAP23 to LDs. Taken together, these data indicate that HCV modulates ARFRP1 to recruit SNAP23 to sites in close proximity to LDs to facilitate viral propagation.

### SNAP23 is required for HCV production

**Figure 6:**
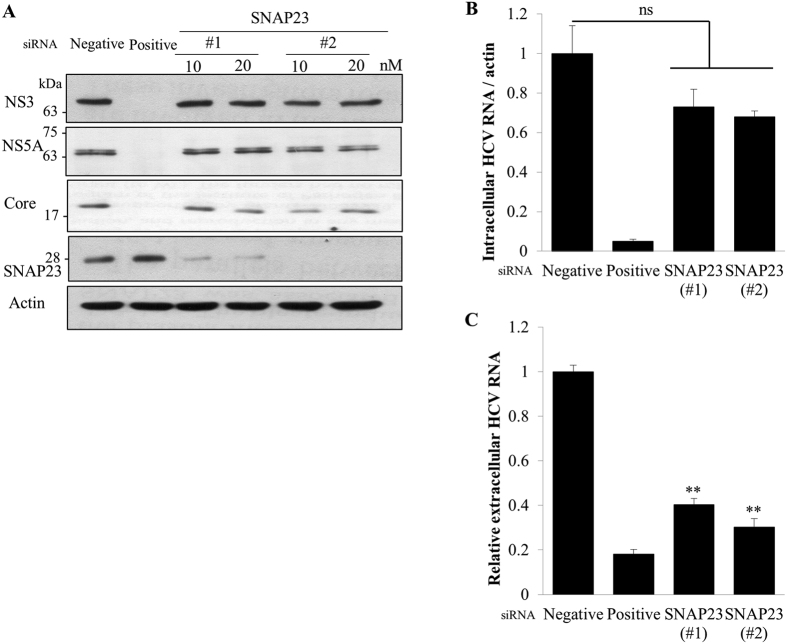
SNAP23 is required for HCV production. (**A**) Huh7.5 cells were transfected with the indicated siRNAs. At 2 days after siRNA transfection, cells were infected with Jc1 for 4 h. At 48 h postinfection, total cell lysates were immunoblotted with the indicated antibodies. (**B**) Huh7.5 cells were treated as described in the legend to Fig. 6A and intracellular HCV RNA levels were quantified by qRT-PCR. (**C**) Huh7.5 cells were treated as described in the legend to Fig. 6A and then extracellular HCV RNAs were analyzed by qRT-PCR. Experiments were performed in duplicate. The asterisks indicate significant differences (**p < 0.01) from the value for the negative control. ns, not significant p value.

It has been previously reported that human cytomegalovirus (HCMV) infection induces the relocation of SNAP23 to the cytoplasmic viral assembly zone and knockdown of SNAP23 inhibits viral production^28^. To investigate whether SNAP23 was directly involved in HCV propagation, Huh7.5 cells were transfected with the indicated siRNAs and then infected with HCV. At 48 h postinfection, HCV protein, intracellular HCV RNA, and extracellular HCV RNA levels were analyzed. As shown in Fig. 6A, silencing of SNAP23 displayed no effect on HCV protein expressions. Similarly, intracellular HCV RNA levels were not affected in SNAP23 knockdown cells (Fig. 6B). However, knockdown of SNAP23 expression led to significant reduction in extracellular HCV RNA level (Fig. 6C), indicating that SNAP23 played a role in late stages of HCV particle production.

## Discussion

Chronic HCV infection often causes steatosis and HCV-induced steatosis is associated with changes in cellular lipid metabolism^4^,^12^,^13^,^14^,^15^. Here, we demonstrated that silencing of ARFRP1, a trans-Golgi network protein involved in LD fusion, resulted in significant reduction in HCV RNA and HCV protein levels, and viral infectivity in HCV-infected cells. We further confirmed that knockdown of ARFRP1 impaired both HCV RNA and protein expressions in HCV replicon cells. These data suggest that HCV may utilize ARFRP1 for both viral replication and LD growth to facilitate virus particle production. To investigate whether ARFRP1 was also involved in another positive-strand RNA virus, we performed siRNA-mediated knockdown experiment using Japanese encephalitis virus (JEV)-infected BHK cells. Protein expression level of JEV was then analyzed by immunoblot analysis using a rabbit anti-JEV NS1 antibody^29^. However, silencing of ARFRP1 displayed no effect on protein expression of JEV (Fig. S3), suggesting that ARFRP1 might be specifically required for HCV propagation.

ARFRP1 selectively interacted with HCV core and NS5A protein. Since ARFRP1 interacted with NS5A stronger than core protein, we further characterized protein interplay between ARFRP1 and NS5A. We demonstrated that ARFRP1 interacted with NS5A through domain I of NS5A. Indeed, LDs were accumulated in replicon cells and HCV-infected cells as well. Moreover, NS5A was partially colocalized with LDs. We also showed that silencing of ARFRP1 abrogated HCV-induced LD accumulation. These data indicate that HCV appropriates host lipid metabolism through modulation of ARFRP1 by NS5A, contributing to liver steatosis which is a pathological hallmark of HCV infection. ARFRP1 is highly expressed in adipose tissue and disruption of ARFRP1 prevents the normal enlargement of LDs and produces an activation of lipolysis^22^. ARFRP1 mediates the transfer of newly formed lipid particles to the storage droplets and hence LD growth is defective in Arfrp1(ad−/−) mice^22^. We showed that protein expression levels of ARFRP1 were higher in hepatoma cell lines than normal liver cells (Fig. S4). We further showed that knockdown of ARFRP1 impaired HCV-induced LD growth. All these data support that ARFRP1 is a host factor involved in the regulation of LD growth in HCV-infected cells.

SNARE proteins mediate fusion of transport vesicles with plasma membrane or target compartments. LDs grow through a fusion process mediated by SNARE proteins, including SNAP23^5^. It has been shown that ARFRP1 acts upstream of SNAP23 and is thereby required for fusion of LDs^5^,^22^. To date, the involvement of SNAP23 in viral propagation has not been fully demonstrated. It has previously been reported that knockdown of SNAP23 inhibits HCMV production^28^. Here, we showed that SNAP23 was recruited to the close sites of LDs in HCV-infected cells, implying that SNAP23 was required for HCV-induced LD enlargement. Indeed, ablation of SNAP23 using siRNA remarkably suppressed HCV production. Our data indicate that LD growth in HCV-infected cells is mediated by cellular ARFRP1. Indeed, we showed that knockdown of ARFRP1 impaired protein relocalization of SNAP23 in close proximity to the LDs in HCV infected cells. We propose that HCV exploits host ARFRP1 for LD growth to make an intracellular milieu suitable for the establishment of persistent HCV infection and replication. Taken together, ARFRP1 is a host proviral factor involved in HCV propagation and may represent a novel anti-HCV therapeutic target.

## Methods

### Plasmids

Total cellular RNAs were isolated from Huh7.5 cells by using RiboEx (GeneAll), and cDNA was synthesized by using a kit (Toyobo) according to the manufacturer’s instructions. Full-length ARFRP1 was amplified by primer sets listed in Table 1. PCR products were inserted into the *Eco*RI and *Bam*HI sites of the plasmid p3XFlag-CMV10 (Sigma Aldrich). siRNA-resistant mutant ARFRP1 (Flag-ARFRP1-SR) was generated by site-directed mutagenesis (Enzynomics) using primers listed in Table 1. siRNA-resistant mutant ARFRP1 contains three silent mutations in the siRNA binding site. Myc-tagged core, NS4B, NS5A, NS5B plasmid were described elsewhere^30^.

### Cell Culture

All cell lines were grown in Dulbecco’s modified Eagle’s medium (DMEM) supplemented with 10% fetal bovine serum and 100 units/ml penicillin/streptomycin in 5% CO_2_ at 37 °C. HCV subgenomic replicon cells were grown as reported previously^30^.

### Antibodies

Antibodies were purchased from the following sources: rabbit-anti SNAP23 antibody from Epitomics; mouse-anti Myc antibody, rabbit anti-ARFRP1 and rabbit anti-GAPDH antibodies from Santa Cruz; mouse anti-Flag and mouse anti-actin antibodies from Sigma-Aldrich; anti-Myc HRP conjugate antibody from Sigma-Aldrich; HCV core, NS3, and NS5A antibodies were described elsewhere^30^.

### RNA interference

siRNAs targeting ARFRP1 (sense, 5′-GUGGAUGGUGAAGUGUGUC-3′; antisense, 5′-GACACACUUCACCAUCCAC-3′), SNAP23 (sequence #1: sense, 5′-CGCAUAACUAAUGAUGCCA-3′; antisense, 5′-UGGCAUCAUUAGUUAUGCG-3′ and sequence #2: sense, 5′-CCAACAGAGAUCGUAUUGA-3′; antisense, 5′-UCAAUACGAUCUCUGUUGG-3′) and the universal negative control siRNA were purchased from Bioneer (Korea). siRNA targeting 5′NTR of HCV (5′-CCUCAAAGAAAAACCAAACUU-3′) was used as a positive control^30^. siRNA transfection was performed using a Lipofectamine RNAiMax reagent (Invitrogen, Carlsbad, CA) according to the manufacturer’s instructions.

### Immunoprecipitation

HEK293T cells were cotransfected with Flag-tagged ARFRP1 and Myc-tagged core, NS4B, NS5A, and NS5B, respectively. Total amounts of DNA were adjusted by adding an empty vector. At 48 h after transfection, cells were harvested and immunoprecipitation assay was performed as we reported previously^30^,^31^. To verify endogenous protein interaction, Huh7.5 cells were infected with Jc1. Total cell lysates harvested at day 4 postinfection were immunoprecipitated with ARFRP1 antibody and bound protein was immunoblotted with rabbit anti-NS5A antibody. Reciprocally, the same cell lysates were immunoprecipitated with rabbit anti-NS5A antibody and bound protein was immunoblotted with an anti-ARFRP1 antibody.

### Immunoblot analysis

Immunoblot analysis was performed as we reported previously^31^. Briefly, equal amounts of proteins were separated by SDS-PAGE and electrotransferred to a nitrocellulose membrane. The membrane was blocked in TBS/Tween (20 mM Tris-HCl (pH 7.6), 150 mM NaCl and 0.25% Tween 20) containing 5% nonfat dry milk for 1 h and then incubated overnight at 4 °C with the indicated antibodies in TBS/Tween containing 1% nonfat dry milk. Following three washes in TBS/Tween, the membrane was incubated with either horseradish peroxidase-conjugated goat anti-rabbit antibody or goat anti-mouse antibody (Jackson ImmunoResearch Laboratories, West Grove, PA) in TBS/Tween for 1 h at room temperature. Proteins were detected using an ECL kit (Amersham Biosciences).

### Focus-forming assay

Huh7.5 cells seeded at 2 × 10^4^ cells in 4-well chamber culture slides (Millipore) were inoculated with serial dilutions of cell culture medium harvested from HCVcc-infected cells. At 2 days after inoculation, indirect immunofluorescence was performed for the presence of intracellular HCV core antigen to determine the numbers of focus-forming units (FFU)/ml as we reported previously^30^.

### Quantification of RNA

Total RNAs were isolated from HCVcc-infected cells, cell culture medium, or replicon cells using RiboEx reagent (Geneall Biotechnology) according to the manufacturer’s instructions. cDNAs were synthesized using cDNA synthesis kit (Toyobo) according to the manufacturer’s instructions. Quantitative real-time PCR (qRT-PCR) experiments were performed using an iQ5 multicolor real-time PCR detection system (Bio-Rad Laboratories, Hercules, CA) as we reported previously^30^,^31^.

### MTT assay

Approximately 4 × 10^4^ Huh7.5 cells seeded on 24-well plates were transfected with the indicated siRNAs. At 4 days after transfection, 3-(4,5-dimethylthiazol-2-yl)-2,5-diphenyltetrazolium bromide (MTT) reagent (Sigma) was added to the cells and incubated at 37 °C for 2 h. Cell viability was determined as we reported previously^32^.

### Immunofluorescence assay

Huh7.5 cells seeded on cover slides were transfected with siRNA constructs. At 48 h after transfection, cells were either mock-infected or infected with Jc1 for 2 days. Cells were washed twice with PBS and fixed with 4% paraformaldehyde and then immunofluorescence assay was performed as we reported previously^31^,^32^. LDs were stained using the BODIPY (439/503) (Invitrogen) as we described elsewhere^33^. Confocal microscopy images were analyzed using the Zeiss LSM 700 laser confocal microscopy system (Carl Zeiss, Inc., Thornwood, NY). Distance between SNAP23 and BODIPY was analyzed by using ImageJ (US National Institutes of Health).

### Statistical analysis

Data are presented as means ± standard deviations (SD). Student *t* test was used for statistical analysis of the data. The asterisks in the figures indicate significant differences (*P < 0.05; **P < 0.01; ***P < 0.001). ns, not significant.

## Additional Information

**How to cite this article**: Lim, Y.-S. *et al*. ADP-ribosylation Factor-related Protein 1 Interacts with NS5A and Regulates Hepatitis C Virus Propagation. *Sci. Rep.* **6**, 31211; doi: 10.1038/srep31211 (2016).

## Supplementary Material

Supplementary Information

## Figures and Tables

**Table 1:** List of primers used in this study.

Primer	Primer sequence	Enzyme site	Purpose
ARFRP1-F	ATG AAT TCA ATG TAC ACG CTG CTG TCG	*Eco*RI	Cloning of human AFRRP1 into p3XFLAG-CMV-10 vector
ARFRP1-R	ATG GAT CCC TAC GTG ATG TCC CTC TG	*Bam*HI	
ARFRP1 resist-mutant-F	GCATCGAGTGGATGGTCAAATGCGTCGTGCGGAATGTGC		Generation of siRNA-resistant mutant of ARFRP1
ARFRP1 resist-mutant-R	GCACATTCCGCACGACGCATTTGACCATCCACTCGATGC		
5′NTR-F	TGAGTGTCGTACAGCCTCCA		Quantitative real-time PCR
5′NTR-R	ACGCTACTCGGCTAGCAGTC		
qActin-F	TGACAGCAGTCGGTTGGAGCG		Quantitative real-time PCR
qActin-R	GACTTCCTGTAACAACGCATCTCATA		
