# Supplementary material for: ADP-ribosylation Factor-related Protein 1 Interacts with NS5A and Regulates Hepatitis C Virus Propagation
Source: Sci Rep. 2016 Aug 23;6:31211. doi: 10.1038/srep31211 (PMC4994002; doi:10.1038/srep31211)

**ADP-ribosylation Factor-related Protein 1 Interacts with NS5A and Regulates Hepatitis C Virus Propagation**

Yun-Sook Lim^1^**†**, Huong T. T. Ngo^1^**†**, Jihye Lee^1¤^, Kidong Son^1^, Eun-Mee Park^1,2^, and Soon B. Hwang^1*^

National Research Laboratory of Hepatitis C Virus and Ilsong Institute of Life Science, Hallym University, Anyang, South Korea^1^, Korea National Institute of Health, Cheongwon-gun, South Korea^2^

**Supplementary Figure Legends**

**Figure S1 ARFRP1 interacts with NS5A stronger than HCV core protein.** HEK293T cells were cotransfected with Flag-tagged ARFRP1 together with either Myc-tagged core or Myc-tagged NS5A expression plasmid. At 48 h after transfection, cell lysates were immunoprecipitated (IP) with an anti-Myc monoclonal antibody, and then bound proteins were detected by immunoblot (IB) analysis using an anti-Flag monoclonal antibody (top panel). Protein expressions of Flag-tagged ARFRP1, Myc-tagged core, and Myc-tagged NS5A were verified by immunoblot analysis with an anti-Flag monoclonal antibody and an anti-Myc monoclonal antibody, respectively (lower panels). Asterisks indicate IgG heavy and light chains.

**Figure S2 ARFRP1 recruits SNAP23 to sites in close proximity to LDs in HCV replicon cells.** (A) Either naïve Huh7 cells or Huh7 cells harboring HCV subgenomic replicon were fixed in 4% paraformaldehyde and immunofluorescence staining was performed by using rabbit anti-SNAP23 monoclonal antibody and TRITC-conjugated donkey anti-rabbit IgG to detect SNAP23 (red). Cells were further incubated with BODIPY (439/503) (Invitrogen) to detect LDs (green). Dual staining showed a partial colocalization of SNAP23 and LDs as yellow fluorescence in the merged image. Cells were counterstained with 4’,6-diamidino-2-phenylindole (DAPI) to label nuclei (blue). (B) Total cell lysates harvested from either Huh7 or Huh7 cells harboring HCV subgenomic replicon were immunoblotted with the indicated antibodies.

**Figure S3** **ARFRP1 is not required for JEV propagation.** BHK cells were transfected with either negative siRNA or various concentrations of ARFRP1-specific siRNA. At 2 days after siRNA transfection, cells were infected with JEV for 4 h. Total cell lysates harvested at 48 h postinfection were immunoblotted with the indicated antibodies.

**Figure S4** **ARFRP1 protein is highly expressed in hepatoma cell lines.** Total cell lysates harvested from each of Huh7.5 cells, HepG2 cells, and Chang liver cells were immunoblotted with the indicated antibodies.


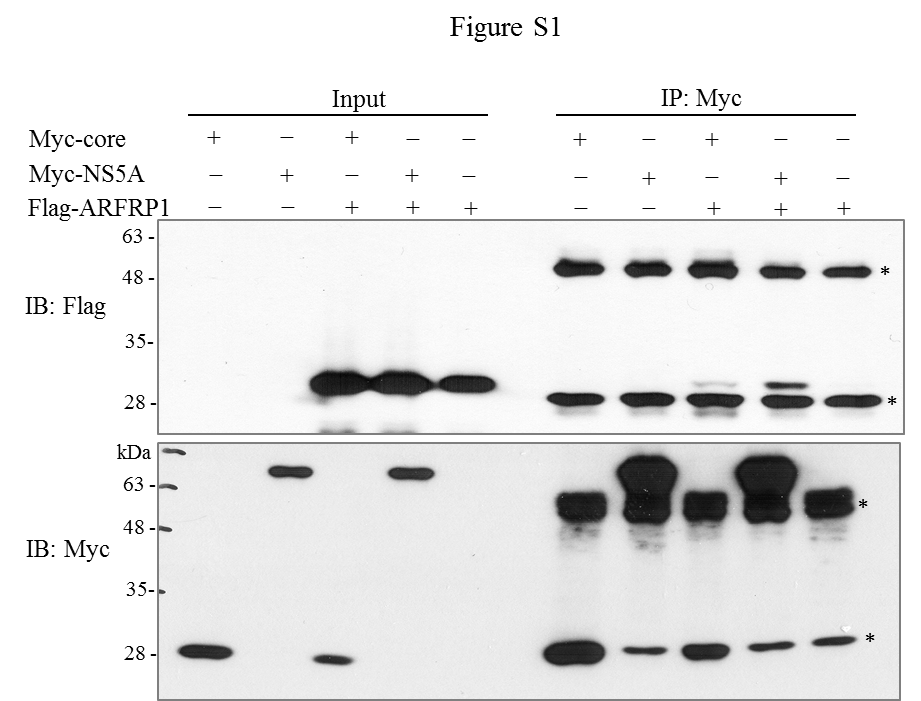


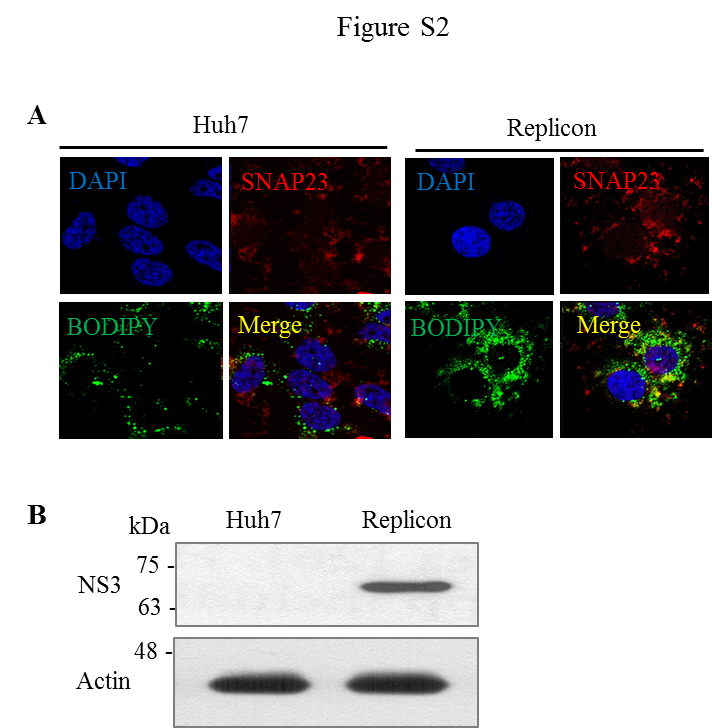


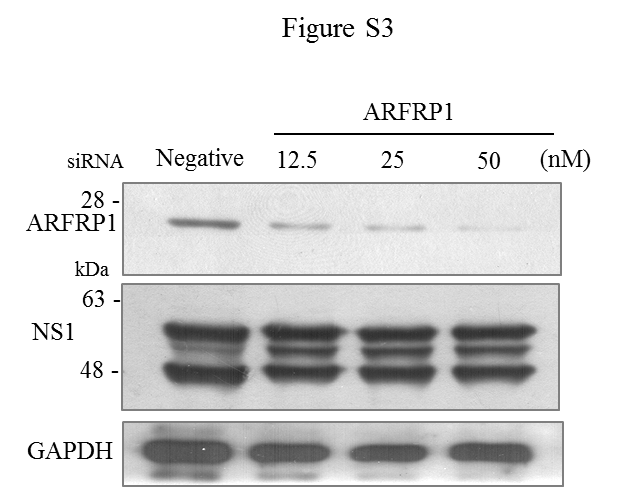


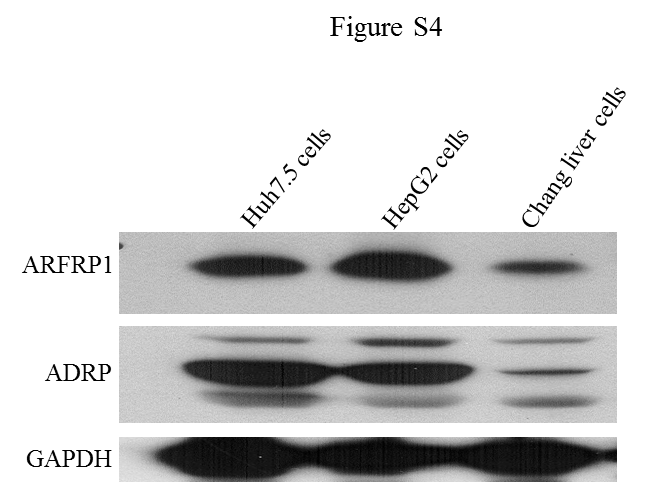

Supplement: Supplementary Information [file srep31211-s1.doc]
